# Distribution and seasonality of rhinovirus and other respiratory viruses in a cross-section of asthmatic children in Trinidad, West Indies

**DOI:** 10.1186/1824-7288-35-16

**Published:** 2009-06-25

**Authors:** Jason Matthew, Lexley M Pinto Pereira, Tressa E Pappas, Cheri A Swenson, Kris A Grindle, Kathy A Roberg, Robert F Lemanske, Wai-Ming Lee, James E Gern

**Affiliations:** 1 Department of Para-Clinical Sciences, The University of the West Indies, St Augustine, Trinidad and Tobago; 2 Department of Pediatrics, University of Wisconsin-Madison (UW-Madison), Madison, WI, USA

## Abstract

**Background:**

Childhood asthma in the Caribbean is advancing in prevalence and morbidity. Though viral respiratory tract infections are reported triggers for exacerbations, information on these infections with asthma is sparse in Caribbean territories. We examined the distribution of respiratory viruses and their association with seasons in acute and stable asthmatic children in Trinidad.

**Methods:**

In a cross-sectional study of 70 wheezing children attending the emergency department for nebulisation and 80 stable control subjects (2 to 16 yr of age) in the asthma clinic, nasal specimens were collected during the dry (*n *= 38, January to May) and rainy (*n *= 112, June to December) seasons. A multitarget, sensitive, specific high-throughput Respiratory MultiCode assay tested for respiratory-virus sequences for eight distinct groups: human rhinovirus, respiratory syncytial virus, parainfluenza virus, influenza virus, metapneumovirus, adenovirus, coronavirus, and enterovirus.

**Results:**

Wheezing children had a higher [χ^2 ^= 5.561, *p *= 0.018] prevalence of respiratory viruses compared with stabilized asthmatics (34.3% (24) versus (vs.) 17.5% (14)). Acute asthmatics were thrice as likely to be infected with a respiratory virus (OR = 2.5, 95% CI = 1.2 – 5.3). The predominant pathogens detected in acute versus stable asthmatics were the rhinovirus (RV) (*n *= 18, 25.7% vs. *n *= 7, 8.8%; *p *= 0.005), respiratory syncytial virus B (RSV B) (*n *= 2, 2.9% vs. *n *= 4, 5.0%), and enterovirus (*n *= 1, 1.4% vs. *n *= 2, 2.5%). Strong odds for rhinoviral infection were observed among nebulised children compared with stable asthmatics (*p *= 0.005, OR = 3.6, 95% CI = 1.4 – 9.3,). RV was prevalent throughout the year (Dry, *n *= 6, 15.8%; Rainy, *n *= 19, 17.0%) and without seasonal association [χ^2 ^= 0.028, *p *= 0.867]. However it was the most frequently detected virus [Dry = 6/10, (60.0%); Rainy = 19/28, (67.9%)] in both seasons.

**Conclusion:**

Emergent wheezing illnesses during childhood can be linked to infection with rhinovirus in Trinidad's tropical environment. Viral-induced exacerbations of asthma are independent of seasons in this tropical climate. Further clinical and virology investigations are recommended on the role of infections with the rhinovirus in Caribbean childhood wheeze.

## Background

Viral respiratory infections are associated with wheezing illnesses and asthma exacerbations in childhood. Rhinovirus (RV), respiratory syncytial virus (RSV), parainfluenza virus (PIV), human metapneumovirus (hMPV) and influenza virus are the major pathogens that are responsible for early wheezing in infancy [1,2]. The Tucson Children's Respiratory Study [3] revealed that lower respiratory tract infection with RSV early in life was associated with persistent wheezing until 11 years of age. More recently Lemanske et al demonstrated that wheezing illnesses due to RV in infancy are the most significant risk factor for the development of persistent wheezing in preschool children [4]

In children above 2 years the rhinovirus is the predominant common cold viral pathogen linked to asthma exacerbations [5]. Viral respiratory tract infections have been the major cause of asthma exacerbations in children [6] with reported prevalence rates of 85% in exacerbations of childhood asthma [7]. Higher rates of respiratory tract infection are associated with a significant increase in asthma admissions observed every autumn (September – November) in the US, Canada, England and Wales [8]. In Canada and France, children who presented to hospitals with asthma exacerbations had a higher prevalence of viral respiratory infections than asymptomatic asthmatic children [9,10].

Studies using conventional and molecular methods to detect respiratory viruses in asthma [6,7], suggest reverse transcriptase polymerase chain reaction (RT – PCR) assays are more rapid, sensitive and specific to detect respiratory viruses compared with conventional techniques such as cell culture. This is especially true for RV, the most common [7] viral trigger for acute exacerbations of asthma.

In the Caribbean, information on respiratory viral infections in childhood asthma is sparse. Using cell culture, Spence et al [11] detected RSV in pharyngeal swabs from 1729 Trinidadian patients with acute respiratory infections from 1964 to 1966 and the majority (90%) of these was in children below four years of age. Outbreaks of RSV infection in Trinidad and Tobago in 1964, 1965 and 1966 occurred during the rainy season in June, September and August respectively. Most studies examining the prevalence and seasonality of respiratory viruses in association with childhood asthma exacerbations have been conducted in temperate climates, which experience seasons which are considerably different in temperature and rainfall than those in the Caribbean. Trinidad has a tropical climate, with 2 seasons; the dry from January to May, and the rainy from June to December. The average temperatures are similar for the two seasons (27.6°C vs. 27.9°C). The major difference between these seasons is the higher average rainfall [Based on average monthly values between January 2000 to December 2005; data obtained through personal correspondence with Trinidad's Meteorological Office] in the rainy season compared with the dry season (203.4 mm vs. 76.5 mm). Being informed by epidemiological data from other geographical locations, we examined if RV was the major viral cause for asthma exacerbations in children in Trinidad, and whether the frequency rates of viral infection differed with seasonality.

## Methods

### Institutional approval

The Ethics Committee of the Faculty of Medical Sciences at The University of the West Indies, and the Health Sciences Institutional Review Board of the University of Wisconsin-Madison approved the study. The research met compliance set out in the Helsinki Declaration and all children's caregivers signed informed consent to participate.

### Subjects

Subjects with acute asthma exacerbations treated in the Accident and Emergency (A&E) departments of the Eric Williams Medical Sciences Complex (EWMSC), the San Fernando General Hospital (SFGH) and the Arima Health Facility (AHF) were recruited, through August 2002 to July 2005 (From February 2004 to January 2005 consecutive periods of industrial action in the facilities did not permit patient recruitment). These facilities provide emergency health care and receive patient referrals from the primary health centres. Stable asthmatics were enrolled as they presented at the asthma paediatric clinic of the AHF where asthma education is offered to patients and their parents/guardians.

### Definitions

Acute asthmatics were defined as asthmatic children who presented to A&E with an asthma exacerbation and required to be nebulised with a bronchodilator to relieve wheezing and/or tightness in the chest and/or difficulty in breathing. Stable asthmatics were non-symptomatic children who had not suffered any acute exacerbation and did not have any need for nebulisation with a bronchodilator in the previous 3 months.

### Inclusion and exclusion criteria

Children between 2–16 years who had received a physician's diagnosis of asthma and who were attending the asthma clinics were eligible for entry to the study. Children less than two years were not included as it is clinically difficult to differentiate wheezing from asthma as opposed to wheezing of other aetiology in very young children. Subjects who presented to A&E and required bronchodilator treatment with a nebuliser, but had not been diagnosed with asthma by a physician were not included. Although subjects made repeat visits to the paediatric clinic and the A&E department, they were entered into the study only on the first occasion. Children with incessant cough and infective conditions were excluded from the study.

### Sample collection

Parents or guardians gave their informed written consent for their child to participate. Consecutive patients were invited to participate in the collection of nasal specimens. Some parents or guardians did not consent as they considered their child was too young (2 – 4 years) for the sampling procedure, and others believed this procedure was a further inconvenience to the child who had just been nebulised. Nasopharyngeal mucus specimens were collected using a #5 Fr feeding tube attached to a 10 ml syringe. Briefly, a maximum volume of 1 ml of sterile saline was administered to the subject's nostril by gently pushing the piston of the syringe; the fluid was then suctioned back into the syringe to obtain the washings of the nasal secretions. The specimens were temporarily stored on wet ice in a cooler and transported within 8 hours of collection to the laboratory (Pharmacology Unit, Faculty of Medical Sciences, The University of the West Indies; Trinidad) where they were separated into 4 aliquots and stored at -70°C until the time of shipping on dry ice to Wisconsin. RNA extraction, reverse transcription of RNA and Respiratory MultiCode assay on all specimens were carried out in the Molecular Virology Section of the Pediatric Allergy/Immunology Research Laboratory at the University of Wisconsin-Madison.

### Molecular viral detection

The Respiratory MultiCode assay (University of Wisconsin-Madison & EraGen Biosciences, Madison, WI, USA) used in this study is a novel high-throughput assay that utilises MultiCode PLx (EraGen Biosciences, Madison, WI, USA) technology. The assay described previously [12] detects 8 distinct respiratory viruses and their collective 160 plus serotypes on 2 detection panels (panels A6B and C3B) portrayed in Table 1. A 96-well assay is completed in approximately four hours and the multiplexing ability of the system allows the possibility for more results from the same sample volume, so that minimal quantities of sample are required. Samples were assayed in duplicate with positive and negative controls for each panel of viral detection.

**Table 1:** Viruses detected by Respiratory MultiCode assay

**Virus**	**Serotypes**	
Rhinovirus	> 101	
Influenza	A, B	
RSV	A, B	**Panel A63B**
Metapneumovirus	A, B	
Parainfluenza virus	I, II, III	
Adenovirus	> 13 serotypes of groups B, C, E	
Coronavirus	OC43, 229E, NL63, SARS	
Enterovirus	> 34	**Panel C3B**

### Statistical analysis

Student's two independent sample t-tests were used to compare differences in the average age between the groups. Chi square tests for independence with the Bonferroni adjustment were used to determine differences in ethnic distribution between the acute and stable asthmatics. Prevalence of viral infections in acute and stable asthmatics and associations of viral prevalence and RV infection with acute asthma and seasons were tested by the chi square (χ^2^) tests for independence. The odds ratios (ORs) with 95% confidence intervals (CI) determined the likelihood of acute asthmatics (a) being infected with a respiratory virus and (b) having a RV infection compared with stable asthmatics. Student's two independent sample t-tests were used to compare differences in the average age between the groups. The Statistical Package for Social Sciences (Chicago, Illinois, version 13.0) was utilised for data analysis. A *p *value less than 0.05 was considered statistically significant.

## Results

### Patient profile

One hundred and fifty (62.8%) of the 239 children interviewed provided nasal specimens for viral analysis. Similar proportions of samples were collected in the dry and rainy seasons from the two groups of children. Boys (*n *= 52, 74.3% vs. *n *= 62, 77.5%) and girls (*n *= 18, 25.7% vs. *n *= 18, 22.5%) were equally distributed (*p *= 0.646) in the acute and stable asthma groups. There was no age difference [*p *= 0.171] between acutely ill (8.0 years, *SD *= 3.5) and stable children (8.8 years, *SD *= 3.2). There was a significant (p = 0.018) difference between acute and stable asthmatics in relation to ethnic distribution, so that children of mixed ethnicity (n = 40, 65.5%) were more frequently stable than were Asian Indian children (n = 11, 35.5%). Equal numbers of nasal specimens were collected in the dry (*n *= 21, 30.0% vs. *n *= 17, 21.3%) and rainy (*n *= 49, 70.0% vs. n = 63, 78.8%) seasons from the acutely ill and stable asthmatic children respectively (p = 0.219]. (Table 2)

**Table 2:** General information of children (*n *= 150) from whom nasal specimens were obtained

	**Acute (*n *= 70)**		**Stable (*n *= 80)**		***P *Value**
	**No**.	**%**	**No**.	**%**	
**Gender**					
Male	52	74.3	62	77.5	0.646
Female	18	25.7	18	22.5	
					
**Age (years)**					
Mean (*SD*)	8.0	(3.5)	8.8	(3.2)	0.171
					
**Ethnicity**					
African	29	41.4	29	36.3	**0.018**
Asian Indian	20	28.6	11	13.8	
Mixed (African & Asian Indian)	21	30.0	40	50.0	
					
**Season specimen was collected**					
Dry	21	30.0	17	21.3	0.219
Rainy	49	70.0	63	78.8	

### Viral detection in acute and chronic asthmatics

**Figure 1:**
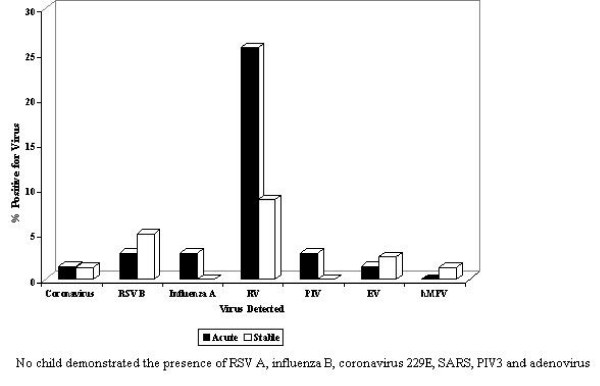
**Viruses detected in acute and stable asthmatic children**.

In children (Table 3) who required nebulisation (n = 24, 34.3%) viral detection was higher (p = 0.018) compared with those (n = 14,17.5%) who were stable. Nebulised children were thrice as likely to be infected with a respiratory virus compared with the stable non-symptomatic children (OR = 2.5, 95% CI = 1.2 – 5.3). Among children in whom viral presence was detected, the rhinovirus was the most frequent and observed in 75% (18/24) of acute asthmatics and in 50% (7/14) of the stable asthmatics. The rhinovirus was the most common viral pathogen detected (p= 0.005), when comparing acutely ill children (25.7%, n = 18) with those who were stable (8.8%, n = 7). The likelihood of an acute asthmatic child being infected with RV was 4 times greater than in a stable asthmatic child (OR = 3.6, 95% CI = 1.4 – 9.3; p= 0.005) (Fig. 1). The RSV B (2.9% (2) vs 5.0%(4)), and enterovirus (1.4% (1) vs 2.5% (2)) were other common viral pathogens detected in acute and stable asthmatic children respectively. Influenza A (2.9% (2), coronavirus OC43 (1.4%(1) and PIV 1 & 2 (1.4%(1) were detected in one child each, who had to be nebulised. Coronavirus NL63 and hMPV were each identified in one child who was stable. Co-infection with > 1 virus was infrequent. Two separate acute asthmatics were positive for Influenza A & RSV B, and Influenza A & RV. One child with non-symptomatic asthma was positive for RV & RSV B. The RSV A, influenza B, coronavirus 229E & SARS, PIV 3 and adenovirus pathogens were not detected in any child.

**Table 3:** Prevalence of viruses in acute and stable asthmatics

**Virus**	**Acute (*N *= 70)**		**Stable (*N *= 80)**	
	**No**.	**%**	**No**.	**%**
Total virus **(*p *= 0.018)**	24	34.3	14	17.5
Virus not present	46	65.7	66	82.5
RV **(*p *= 0.005)**	18	25.7	7	8.8
RV/Total virus	0.75	75.0	0.50	50.0
RSVA	0	0.0	0	0.0
RSV B	2	2.9	4	5.0
Influenza A	2	2.9	0	0.0
Influenza B	0	0.0	0	0.0
Coronavirus OC43	1	1.4	0	0.0
Coronavirus NL63	0	0.0	1	1.3
Coronavirus 229E	0	0.0	0	0.0
Coronavirus SARS	0	0.0	0	0.0
EV	1	1.4	2	2.5
PIV 1	1	1.4	0	0.0
PIV 2	1	1.4	0	0.0
PIV 3	0	0.0	0	0.0
hMPV	0	0.0	1	1.3
Adenovirus	0	0.0	0	0.0
Co-infection with 2 viruses	2	2.9	1	1.3
-Influenza A & RSV B	1	1.4	0	0.0
- Influenza A &RV	1	1.4	0	0.0
- RV & RSV B	0	0.0	1	1.3

### Seasonal distribution of viruses detected

**Figure 2:**
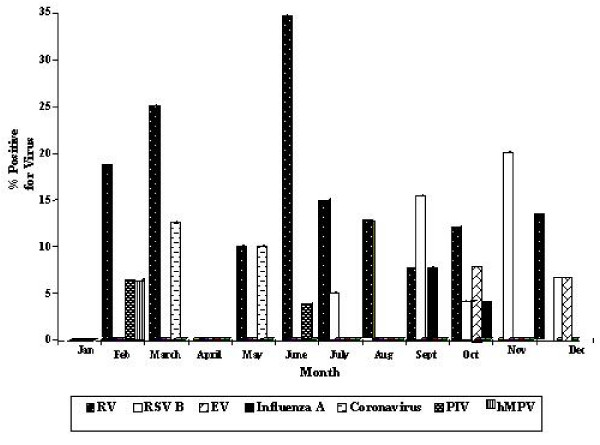
**Year round prevalence of respiratory viruses in asthmatic children**.

**Figure 3:**
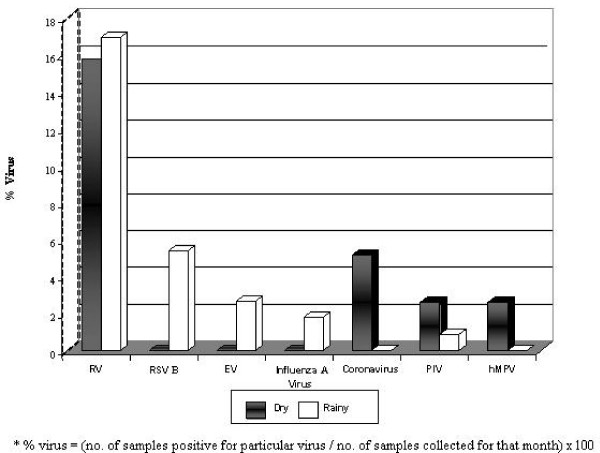
**Respiratory viruses detected in the dry and rainy seasons in children with asthma**.

Evidence of viral infection was present throughout the year and highest in February (31.4%), March (37.5%) and June (38.5%) but was not detected in January and April (Fig 2). Viral detection fell steeply from June to July (20.0%) and further in August (12.5%). A sharp rise in viral identification was observed from August to September (30.8%), and appeared to be sustained from October (28.0%) to December (26.7%). Viral prevalence did not differ (*p *= 0.872) in the dry (*n *= 10, 26.3%) and rainy (*n *= 28, 25.0%) seasons (Fig 3). Coronavirus OC43 or NL63 (5.3%(2)) and hMPV (2.6%(1)) were detected only in the dry season, whereas RSV B (5.4% (6)), enterovirus (2.7% (3)) and influenza A (1.8%(2)) were identified only in the rainy season. Viral pathogens detected throughout the year were PIV2 (Dry, 2.6% (1)), PIV1 (Rainy, (0.9% (1) and RV (Dry, 15.8% (6); 17.0% (19). The RV was not associated (p = 0.867) with any particular season, and in both seasons [Dry = 6/10, (60.0%) Rainy = 19/28, (67.9%)] it was the most frequently prevalent viral pathogen detected.

## Discussion

This study demonstrates that 34.3% of children in Trinidad who are wheezing and need to be nebulised have a viral infection. The viral prevalence in children with asymptomatic disease is 17.5%. Our observations on the viral association with paediatric asthma are the first from the Caribbean region and Trinidad in particular where childhood asthma inflicts a burden on the health system [13]. The association of viral infections with exacerbations of wheeze in children with asthma has been reported in temperate climates with higher rates compared with our findings. In Britain viruses were detected in 85% of children with episodes of wheeze and symptoms of asthma, and rhinovirus was the most frequent virus that was detected [7]. Rakes et al studied infants and children with severe wheezing attending the emergency department in Virginia, USA, and identified rhinovirus in 71% of those children [5]. However, in France Freymuth et al [6] observed lower rates of rhinovirus as the associated pathogen in 46.9% of nasal aspirates in hospitalized children who had a severe attack of asthma. As far as these authors are aware viral infections in exacerbations of asthma have not been reported from countries which enjoy warm tropical climates.

In Brazil Camara et al [14] investigated viral infections as risk factors for acute wheezing in children between 0 – 12 years and reported RV was not associated with wheezing and did not appear to trigger asthma exacerbations. These findings, they explain, may be attributed to different immunologic responses to RV by the asthmatic population in Brazil compared with other populations studied. They suggest the RV serotypes circulating in the tropics may differ from those in temperate climates, so that the PCR methodology used would not have detected these serotypes. The tropics have higher year-round temperatures which may limit RV replication providing an explanation for the low prevalence rates in this climatic zone compared with those reported from temperate climates. Viral detection rates between exacerbations when children are asymptomatic are very low, to the order of just 3–12% [15]. Polarized viral infection rates present in temperate climates may result from the wide differences in temperature these regions experience, as opposed to the tropics which enjoy relatively continual year-round warmer temperatures, which probably sustain moderate viral presence throughout the year. It may be worth speculating that the viral trigger for exacerbations of asthma may manifest more readily when temperatures are low.

Earlier we observed that children requiring nebulisation were thrice as likely to have a virus infection as did stable children [16]. The common cold/'virus' was a frequently reported trigger of paediatric asthma and children who were nebulised were more likely to have cough, fever or sore throat than did stable children, in the week before they presented at the clinic. Common cold viruses, particularly RV have been implicated as a major trigger for asthma exacerbations. Respiratory tract infections which include the common cold have been associated with an increase in asthma symptoms, hospitalisations and exacerbations [17-19]. Despite the significant association between rhinovirus and exacerbations of asthma observed in the children we studied, the prevalence (25.7%) compares poorly with reported rates from France [7], Britain [7] and the USA [5]. Children may have presented to hospital, some days after their initial symptoms, when it might no longer be possible to recover the potential virus that was provocative of the initial illness. Specimens collected in Trinidad, stored, and shipped to another location for processing may have undergone viral attrition during storage, delays in trans-shipment of samples from Trinidad to Wisconsin and thawing of frozen samples.

Increased reactivity during RV infection may be associated with greater changes in airflow limitation, as asthmatic subjects do demonstrate a variable clinical and pathological response [20,21]. The central prevalence of RV among other respiratory viral pathogens isolated signals that in Trinidad's tropical climate it appears to be the major viral trigger for paediatric asthma exacerbations just as reported in the temperate climatic zones. To these authors' knowledge, this is the first report linking RV as a major viral trigger for paediatric asthma in the tropics. These findings need to be further explored in longitudinal studies over changing seasons in Trinidad.

In the underlying sample 70% of children required nebulisation in the rainy season compared with just 30% of asthmatics in the dry season. Other reports from Trinidad [13,22] Barbados [23] and Mexico City [24] also observed higher disease exacerbations in the rainy season. Even though the overall viral prevalence was higher in the rainy season the difference was not of statistical significance. However the distribution of viruses throughout the year in the present study seems to correspond with reported admission rates in children with asthma in Trinidad [25,26]. Admission rates for asthmatic children in Trinidad were reported to be lowest in April [25], relatively low in January and at the lowest point in August in the study period of January to December 1997 [26]. The absence of viral prevalence in January and April, with a nadir in August (12.5%) in the present study offers an explanation for these earlier reported observations. The sharp rise in viral detection in September, which remained relatively high for the rest of the year till December correlates with Trinidad's hospital admission data [25] and puts forward justification for the back-to school September peak observed in Trinidad [26] as in Mexico [24], Canada [27], the UK [28] and the US [29].

We detected RSV exclusively in the rainy season (June – December). The 1964–1966 RSV outbreaks in Trinidad began in the respective months of June, September and August in the rainy season [11]. A retrospective study from 1982 to 1997 in Malaysia [30], showed a clear association between RSV infection rates and the rainy season. In the USA, RSV is most prevalent during the winter months, particularly in January or February [31]. Whether the RSV A strain causes more severe symptoms is controversial [32,33], but only the B subtype RSV strain was detected in children we studied. A low prevalence rate was not unexpected in the children sampled who were between 2 – 16 years as RSV infection is highest below the age of 2 years [34]. The significant lower prevalence of other respiratory viruses in the asthmatic children has been reported in other investigations [6,7,14].

The importance of RV infections in acute asthma has been seen in other studies where it was associated with 71%–85% [5,7] of exacerbations, and was also a recognized pathogen for the lower airway [35]. It may be worthwhile to investigate children who wheeze below 3 years for the presence of RV infection. Children with RV-induced wheeze in infancy were 4 times more likely to develop asthma by the age of 6 years than those in whom wheezing was associated with other viruses [36]. Researchers at the University of Wisconsin followed high-risk children (with either one or both parents having had allergies or asthma) from birth to six years in the Childhood Origins of Asthma (COAST) study and evaluated them for the presence of specific viruses during wheezing illnesses. Children who had RV and wheezed during the first year of life were nearly three times as likely to have asthma at six years of age, whereas those children with RSV who wheezed did not have an increased asthma risk. The authors concluded that 'wheezing RV illnesses occurring at any time during the first three years of life were associated with a nearly 10-fold increase in asthma risk at six years', making this viral trigger a significant predictor of asthma development in high risk children. [37]. According to Johnston and co-workers [7] viral infection of the upper airways by common cold viruses frequently triggers a response in the lower airways leading to prolonged morbidity, particularly in those subjects who have significant pre-existing airway disease. Early detection of RV-induced wheeze in very young children assumes clinical importance and encourages close monitoring because of the increased risk of developing early childhood wheeze.

The major viral trigger for paediatric asthma in Trinidad was RV which was not associated with seasonality, suggesting that the virus presents a year round risk factor for asthmatic children in Trinidad. In an earlier study we found children with asthma exacerbations reported symptoms of cold, fever and sore throat that they attributed to a 'cold' in the seven days before visiting the emergency room [16]. These symptoms may have been due to an allergic response mistaken for an infection, or may have been manifestations of a true viral infection. We did not examine children for associated allergic rhinitis which may have contributed to a combination of viral infection and allergen exposure on airway physiology and inflammation. Allergens and viruses may act synergistically to exacerbate asthma in sensitized patients exposing them to an increased risk of exacerbations. In asthmatics even a mild cold is sufficient to induce exacerbations of wheeze, and serious life-threatening asthma attacks are quite likely to occur in association with a severe cold. Spread of viruses through the community to susceptible individuals may be the single most important cause of sustained exacerbations of asthma in children. We suggest strategies to reduce the impact of asthma exacerbations should include interventions directed at viruses, as well as at reducing allergen exposure. Factors other than allergens and viruses, such as air pollution and cigarette smoking should also be examined, in addition to unavailability of and non-compliance with treatment, which was observed in asthmatics attending the Chest Clinic in Trinidad [38]. Caregivers of asthmatic children must be educated to recognize the pro-dromal symptoms associated with viral upper respiratory tract infections so that prompt and aggressive anti-inflammatory controller medication can be initiated.

## Conclusion

As in temperate climates RV presents a risk factor as the major viral trigger for asthmatic children in Trinidad. Viral-induced exacerbations of asthma are independent of seasons in this tropical climatic zone. This study provides a basis for further clinical and virology investigations on the role of RV infections in Caribbean childhood wheeze. As an important clinical implication early prevention and treatment of exacerbations of wheeze in children should focus on upper respiratory tract viral infections. Such strategies may assist to optimize healthcare in paediatric asthma and reduce its burden in this tropical region.

## Abbreviations

(RSV): Respiratory synctial virus; (RV): Rhinovirus; (A&E): Accident and Emergency; (EWMSC): Eric Williams Medical Sciences Complex; (SFGH): San Fernando General Hospital; (AHF): Arima Health Facility; (hMPV): human Metapneumovirus; (SARS): Severe Acute Respiratory Syndrome; (PIV): parainfluenza virus; (PCR): polymerase chain reaction; (CI): confidence intervals.

## Competing interests

The authors declare that they have no competing interests.

## Authors' contributions

JM did the sample collection, laboratory and statistical analysis and prepared the draft of the manuscript. LMPP conceived, designed, supervised and co-ordinated the study and prepared the final manuscript. TEP, CAS, KAG, and KAR participated with the viral assays and co-ordinated the ethical approval. WML standardized the methodology on the high-throughput assay and trained on its application. JE and RFL were senior supervisors at the University of Wisconsin who critiqued the analysis, data, results, and manuscript. JE lent significant personal support and encouragement during the analysis and the conclusions. All authors approved the final manuscript.
